# Efficacy of a Group Psychoeducation Treatment in Binge Eating Disorder: An Open-Label Study

**DOI:** 10.3389/fpsyt.2022.822282

**Published:** 2022-04-26

**Authors:** Silvia Liquori, Giovanni Faidutti, Marco Garzitto, Luana Saetti, Monica Bendotti, Matteo Balestrieri

**Affiliations:** Unit of Psychiatry, Department of Medicine (DAME), Centre for Eating Disorders, University of Udine and Friuli Centrale Health-University Trust (ASUFC), Udine, Italy

**Keywords:** Binge Eating Disorder (BED), body mass index, body image, self-esteem, psychoeducation, group treatment

## Abstract

**Aims:**

To evaluate the effectiveness of a multidisciplinary group psychoeducation treatment (GPT) in patients with Binge Eating Disorder (BED).

**Methods:**

We designed an open-label efficacy study that included a population of 45 patients diagnosed with BED. A measure of eating attitudes and associated psychological constructs was obtained through the use of the self-report instruments Eating Disorder Inventory (EDI-3), Binge Eating Scale (BES) and Body Uneasiness Test (BUT). The Symptom Checklist-90 (SCL-90) was also administered to assess general psychopathology. All participants participated in 12 weekly group psychoeducational treatment meetings, 8 of which were conducted by mental health professionals, and a second module of 4 meetings, oriented on health and nutrition education topics, conducted by a dietician.

**Results:**

At the end of treatment, patients showed significant improvements in body mass index (BMI) and binge eating. Paired *t*-tests showed significant differences at *p*-value < 0.05 in all eating disorder risk scales and for most of the general psychological scales related to eating disorders. In addition, patients experienced an improvement in their perception of overall body image, a decrease in concerns about physical appearance and less body image avoidance. Finally, results showed that psychoeducation treatment was associated with significant improvements in interpersonal sensitivity.

**Conclusions:**

The results of this study may indicate that significant short-term improvements can be achieved through a psychoeducation group for BED patients. Although limited by the lack of a control condition, this study adds to a growing body of evidence with promising results, setting the stage for further case-control studies of BED treatment.

## Introduction

The fifth edition of the Diagnostic and Statistical Manual of Mental Disorders (DSM 5) introduced Binge Eating Disorder (BED) as a formal diagnosis (1). BED is an eating disorder characterized by recurrent episodes of ingestion of large amounts of food in a short period of time (binges), accompanied by a sense of loss of control during the episode, without the use of compensatory techniques. To be diagnosed with BED, binge episodes must occur at least once a week for three months and at least three of the following symptoms must be present: (a) eating much faster than normal, (b) eating until you feel unpleasantly full, (c) eating large amounts of food without being hungry, (d) eating alone because of embarrassment about the amounts of food eaten, and (e) feeling disgusted with oneself, depressed or very guilty after each episode.

BED is the most common eating disorder: lifetime prevalence estimates average 1.9% across surveys conducted in all over the world (2). It is often related to obesity, even if the latter is not included in the diagnostic criteria: 30.7% of individuals with BED are overweight, while 32.8% suffer from obesity (2, 3). Due to excessive calorie intake, BED patients have an increased risk of medical complications, such as metabolic syndrome, type 2 diabetes, hypertension and cardiovascular disease (4). In addition, BED often has an impact on sufferers' quality of life and is associated with psychiatric comorbidities, particularly mood and anxiety disorders, alcohol and drug use disorders (5).

Psychiatric comorbidity in patients with BED predicts worse psychopathology than the eating disorder alone and a higher frequency of binge eating during treatments (6). Different risk factors have been identified for BED, including environmental factors and individual factors (3). Since BED is a complex disorder affecting both mind and body, the evaluation must be multidimensional, encompassing psychiatric, psychological, dietician- nutritionist and internist component. Among the psychotherapies proposed for BED there are the cognitive behavioral therapy (CBT), the interpersonal psychotherapy (IPT), and the behavioral weight loss treatment (BWL) (7–9). A review of the evidence-based worldwide clinical guidelines for eating disorders concluded that cognitive-behavioral therapy (CBT) was consistently recommended for BED by all guidelines, followed by guided or unguided cognitive-behavioral self-help treatment and interpersonal psychotherapy (IPT). An explicit recommendation for psychodynamic therapy was made by the German guidelines only (10).

Over the years, research has focused on the development of cheaper treatments with the aim of making them more easily accessible. For this reason, several studies have evaluated the effectiveness of psychoeducation interventions for people with anxiety, depression, bipolar disorders, schizophrenia and other mental health disorders (11–13). Of these, only few were addressed at the treatment of patients with BED (14, 15). Psychoeducation is a treatment that promotes educational interventions on physical illnesses and psychiatric disorders, with the aim of making the patient informed and able to manage the mechanisms that maintain the illnesses. This treatment aims to respond to the patient's needs to familiarize with their disorder and to learn about the intervention techniques relevant to the management of their disease. The goal of psychoeducation is to prevent the worsening of the disease and help the patient reach their maximum state of health (16). Furthermore, the patient could participate in the treatment through active collaboration in order to cope with their physical and psychological health by cooperating with the healthcare personnel. As opposed to individual treatment, group treatment allows a reduction in the cost of treatment since more patients are treated in a limited amount of time. The group treatment offers several advantages: patients feel supported by sharing common problems, they can explore and discuss emotions in a setting where they can be accepted and understood (17). Therefore, they develop new life skills such as a greater self-esteem, the ability to socialize and they reduce their sense of social isolation (18).

In our research we applied a 12-weeks multidisciplinary Group Psychoeducation Treatment (GPT) to explore its feasibility and effectiveness in patients with BED. Our clinical group program used a psychoeducation model that incorporated instructional lessons and behavioral strategies through multidisciplinary professional support (i.e., psychiatric/psychological and nutritional). The aims of the treatment were to improve the patients' understanding of the disease, make them understand the factors that cause and maintain binge eating behavior, explore basic emotions and learn emotion regulation strategies, and gain knowledge about healthy eating and lifestyle behaviors in order to improve both their treatment and their health.

## Methods

### Participants

The study was conducted on patients with BED recruited consecutively from 2018 to 2021 at the outpatient service and day clinic of the Center for Eating Disorders of the Local Health Authority in Udine, Italy.

All participants were referred to this service to undergo a psychiatric evaluation to assess the possible presence of an eating disorder. When a diagnosis of BED was ascertained, patients were asked to take part in a Group Psychoeducation Treatment (GPT) program. To be eligible, participants had to meet the following criteria: ability to provide written informed consent, sufficient language skills to complete the self- administered questionnaires. The intervention required that patients following the GPT were not receiving other weight loss treatments (weight-loss medication, structured diet, weight counseling, etc.) while attending the group. Participants didn't receive any form of compensation for the completion of this program. Dropout was defined as early termination of treatment (i.e., missing more than 20% of the sessions). A detail of the socio demographic characteristics of this sample is detailed in Table 1.

**Table 1:** Characteristics of the sample.

**Characteristics**	***N* (%) or mean**
Females	39 (86.7%)
Age (years)	47.1 ±10.7
Married	27 (59.0%)
Unemployed	13 (28.9%)
Education	
Primary/secondary school High school Degree	16 (37.8%) 19 (44.5%) 9 (20.0%)
Duration of disorder (years)	
<4 4–7 8–15 >15	3 (8.6%) 10 (20.0%) 17 (37.1%) 15 (34.3%)
Comorbid medical conditions	
Problems with glycaemia Heart-associated problems	10 (22.2%) 10 (22.2%)
Obesity classes	
Normal Overweight Class I Class II Class III	4 (8.9%) 2 (4.4%) 8 (17.8%) 13 (28.9%) 18 (40.0%)
BMI (mean)	37.1 ±7.2
Binges per week	
1–2 3–4 5–8	17 (37.1%) 20 (45.7%) 8 (17.2%)
Drugs	
Antidepressants Antipsychotics Anxiolytic-hypnotics Other drugs Any	18 (40.0%) 5 (11.1%) 2 (4.4%) 7 (15.5%) 21 (46.6%)
Voluptuary substances	
Tobacco Alcohol	6 (13.3%) 4 (8.8%)

### Group Intervention

The treatment protocol consisted of 12 90-min weekly sessions of group treatment. The program included two teaching modules: the first consisted of eight sessions (subdivided in four parts) conducted by a team consisting of a psychiatry resident and a clinical psychologist (mental health team), appropriately trained for the task by a group psychoeducation expert. The second module included four health education lessons, given by a professional dietitian accompanied by the mental health team.

Treatment groups comprised a small number of patients, from 6 to 11 individuals, allowing each participant to be involved to some extent in the interactive parts of the program (i.e., role-play, reading of one's diary at the beginning of each session). The leaders were trained to allow and facilitate maximum interaction among the participants, while maintaining a space free of criticism and stigma.

The program was designed to progress from the most basic concepts to gradually more complex and comprehensive tasks, so that everything taught from the beginning was summarized and integrated in subsequent meetings. As each lesson was linked with the previous ones, the group could not be implemented with new patients and had limited members, so that the content could be deepened. Once a person completed the treatment it was not allowed for them to participate again to a different GPT. They would be re-evaluated by the psychiatrist, who would then decide how to continue the path of care.

### Psychoeducation Module (Mental Health Team), Eight Sessions

From the first session, participants were required to write a food diary each day, which included sections on recording their emotions and thoughts when eating and assessing their sense of hunger and fullness. The diary was not intended as a tool to assess or control participants' eating patterns, so it was forbidden to use precise measures of food intake or count calories. Instead, it was used to encourage self-monitoring and served during the 12 weeks to implement the lessons learned and for daily practice of the skills taught.

The contents of the psychoeducation sessions of this module were divided as follows (Table 2):

- The first part consisted of two psychoeducation sessions focused on how to use the food diary and on creating a conceptual background of the disorder: defining a binge, distinguishing between biological and emotional hunger, all-nothing thinking, low self-esteem. It was made clear that the aim of the intervention was not to lose weight. In addition, some less obvious features of the disorder were emphasized, namely how drastic diet, rapid weight loss, overestimation of body image and shape can be characteristic of BED as binges and overweight/obesity (19).
- The second part included the third and fourth sessions focused on teaching the cognitive model of emotion, taking from appraisal theory (20), and the cognitive emotion regulation strategy of reappraisal (21). The intent of this module was to address the emotion regulation issues that research has shown to be linked to binge eating behavior (22).
- The third part included the fifth and sixth sessions, which addressed interpersonal stressors, how they can trigger binges and how these stressors can be relevant to the maintenance and recurrence of dysfunctional behavior (23). Patients were taught how to identify passive and/or aggressive behavior in themselves and others and how to practice more functional and assertive communication strategies as an alternative to rigid and automatic responses.
- The fourth part included the last two sessions of the mental health group, which were dedicated to the integration among the different topics outlined during the program, involving participants in role-plays, asking them to simulate everyday situations, in order to experiment assertive communication techniques. All newly learned skills were framed in a problem-solving perspective, to help identify and circumscribe the problem faced. The scope was to maximize the translation of all knowledge and skills to the real world.

**Table 2:** Group program schedule.

**Week**	**Module**	**Principal professionals**	**Content of the session**
1	Psychoeducation	Mental health	Introduction
2	Psychoeducation	Mental health	BED: Definition and Mechanisms
3	Health Education	Dietician	Water and Fiber
4	Psychoeducation	Mental health	The Emotional Alphabet
5	Psychoeducation	Mental health	Emotion Regulation
6	Health Education	Dietician	Carbohydrates
7	Psychoeducation	Mental health	Communication & Assertiveness
8	Psychoeducation	Mental health	Assertive Communication: Practice Session
9	Health Education	Dietician	Proteins & Fats
10	Psychoeducation	Mental health	Problem Solving
11	Psychoeducation	Mental health	Problem Solving: Practice Session
12	Health Education	Dietician	Practical Tools Healthy Eating

### Health Education Module (Dietitian With Participation of the Mental Health Team), Four Sessions

Health education lessons took place after each of the 4 different parts of psychoeducation. The aim of this module was to provide participants with useful knowledge and information about the main macronutrients to be included in a balanced meal, the Mediterranean diet, learning behavioral techniques that can facilitate lifestyle changes and reduce the likely of binges. These included tips on how to shop for food and groceries, learning to plan ahead for the meals throughout the week, how to avoid and recognize junk food, and how this type of food can easily break our innate mechanism to regulate food intake.

### Assessment

All patients undertook a multidisciplinary assessment for BED, including psychiatric assessment using DSM-5 criteria, psychological profile, nutritional and dietary counseling and blood tests to assess metabolic and endocrine functioning.

A set of questionnaires was administered to all participants at the beginning of the treatment (t0) and after 12 weeks (t1). The tests used were:

- Eating Disorders Inventory, third version (EDI-3): this is a self-administered questionnaire aimed at the clinical evaluation of symptoms associated with eating disorders. EDI-3 consists of 91 items divided into 12 dimensions: three scales of eating disorder risk (i.e., DT, Drive for Thinness; B, Bulimia; BD, Body Dissatisfaction) and nine general psychological scales (LSE, Low Self-Esteem; PA, Personal Alienation; II, Interpersonal Insecurity; IA, Interpersonal Alienation; ID, Interoceptive Deficits; ED, Emotional dysregulation; P, Perfectionism; A, Asceticism; MF, Maturity Fear). The questionnaire includes also six composite scales derived from the recombination of scores (EDRC, Eating Disorder Risk; IC, Ineffectiveness; IPC, Interpersonal Problems; APC, Affective Problems; OC, Overcontrol) (24). In this work, we referred to percentile scores from the Italian standardization of the questionnaire (25). A T-score of 85 was used as cut-off for clinically significant problems and a score of 70 for the borderline level of difficulties. Cronbach's α coefficients of the Italian version indicated an acceptable-to-good internal consistency (0.72 < α <0.94) (25), with similar results also in Italian patients with BED [α from 0.62 to 0.82; (26)].
- Binge Eating Scale (BES): this is a self-assessment questionnaire that allows to evaluate the emotions and behaviors related to binge-eating episodes. It consists of 16 assertions describing different behavioral (e.g., eating habits) and emotional (e.g., guilt, shame) aspects relating to nutrition, to which the compilers give a score of agreement. The unidimensional score obtained ranges from 0 to 46 and a score ≥17 has been proposed as an optimal cut- off to identify BED (27, 28). In an Italian sample of patients with BED, an α of 0.88 was reported (29).
- Body Uneasiness Test (BUT): this is a self-administration test aimed at assessing body image. The test consists of two parts: the BUT-A which includes 34 items and measures: Weight Phobia (WP); Body Image Concern (BIC); Avoidance Behaviors (AB); Compulsive Self-Monitoring (CSM); Depersonalization (D), a sense of detachment and depersonalization toward one's own body. The BUT-A also includes an overall score, the Global Severity Index (GSI). The second part, BUT-B, includes 37 items and it focuses on concerns about specific parts or functions of the body (giving two symptom scores: PST, Positive Symptom Total; PSDI, Positive Symptom Distress Index). Current data has been compared with normative sample collected in Italy (30). We considered a z-score above 1.5 as indicative of clinically relevant symptoms. The Cronbach's α coefficients for the Italian version ranged from 0.64 to 0.89, with all the subscales but one (BUT-B VII, a two-item factor not included in our study) showing α > 0.70 (31). In an Italian sample of patients with BED, α was 0.91 for the BUT-GSI and 0.89 for the BUT-PST (32).
- Symptom Checklist 90, revised version (SCL-90-R): this is a self-administered 90-item questionnaire that evaluates a broad spectrum of internalizing and externalizing symptoms (33, 34). The questionnaire provides 10 specific scores (i.e., SOM, Somatization; O-C, Obsessive-Compulsive; I-S, Interpersonal Sensitivity; DEP, Depression; ANX, Anxiety; HOS, Hostility, PHOB, Phobic Anxiety; PAR, Paranoid Ideation; PSY, Psychoticism; SLEEP, Sleep problems) and an overall score (GSI, Global Severity Index). The SCL-90-R was here used to measure overall well-being, especially in the measurement of outcome. The instrument has been widely used in eating disorders (35, 36) and in BED (37). The Italian version of the checklist achieved a satisfactory internal consistency, with Cronbach's α = 0.68–0.87 for the single scales and 0.97 for the GSI score (34). In an Italian sample of patients with BED, the GSI internal consistency was good, with α = 0.84 (38).

### Statistical Analysis

Group differences before the intervention were analyzed using between-group Welch-corrected *t*-test, or Mann-Whitney's test (as non-parametric alternative for heteroskedastic distributions). We also used single-sample *t*-tests and Mann-Whitney's test to evaluate differences with the normative samples of EDI-3 and BUT-A. For categorical measures, Fisher's exact test was used. The analyses on the effects of the intervention were carried out with repeated-measures *t*-tests or Wilcoxon's test (for continuous measures) and McNemar's χ^2^-test (for categorical measures). Cohen's d for paired samples (with 95% confidence interval) was adopted as a measure of effect-size and it was conventionally considered to be of medium size for 0.5 ≤ |d| <0.8, of small size for 0.2 ≤ |d| <0.5, and negligible for |d| <0.2.

To examine the possible effect of participants' characteristics on the outcome, linear mixed-effects models were used for the repeated assessments (with participants as random factors). A series of possible covariates/confounders were included as fixed effects in the models, testing their statistical significance with the maximum likelihood method. Participants' sex, education, occupation, marital status, use of tobacco and alcohol, medical comorbidities, pharmacological and psychopharmacological treatments were considered. Pre-treatment frequency of binge eating, being in state of obesity, and disorder duration were also evaluated. Statistical significance was set at α = 0.05. In pre-post comparisons, one-tail hypotheses were tested, as the expectation was a decrease for all outcomes considered. In order to control for type I errors without losing too much statistical power, the correction method for independent multiple comparisons based on the False Discovery Rate (FDR) of Benjamini and Hochberg was used. In the correction, we considered 39 independent measures (i.e., BMI, BES, 18 EDI-3 scales, six BUT-A scales, two BUT-B scales, 11 SCL-90-R).

All analyses were conducted with R, version 4.1.1 (39).

## Results

The initial sample consisted of 63 patients; 18 (28.5 %) failed to complete at least 80% of the sessions and were not included in the analyses. The final sample, therefore included 45 patients, six of whom (13.3%) were males (Table 2). Participants had a mean age of 47.1 ± 10.7 years (ranging from 21 to 67). Most were married, and 28.9% were unemployed. Medical comorbidities included impaired blood glucose or diabetes (22.9%) and cardiovascular problems (also 22.9%).

In 71.4% of participants the duration of disorder was more than 8 years. 86.7% of the sample had obesity (BMI > 29.9), in majority of class III. At the first assessment, the mean BMI was 37.1 ± 7.2 kg/m^2^ (20.4 to 48.9). The mean number of weekly binge-eating episodes was 3.3 ± 1.7, with 17.2% of participants having more than four binges per week. Psychotropic drugs use was reported by 47.7% of cases, antidepressants being the most commonly prescribed (40.9%). Tobacco users accounted for 14.3%, while alcohol consumption was less frequent (8.6%).

**Figure 1:**
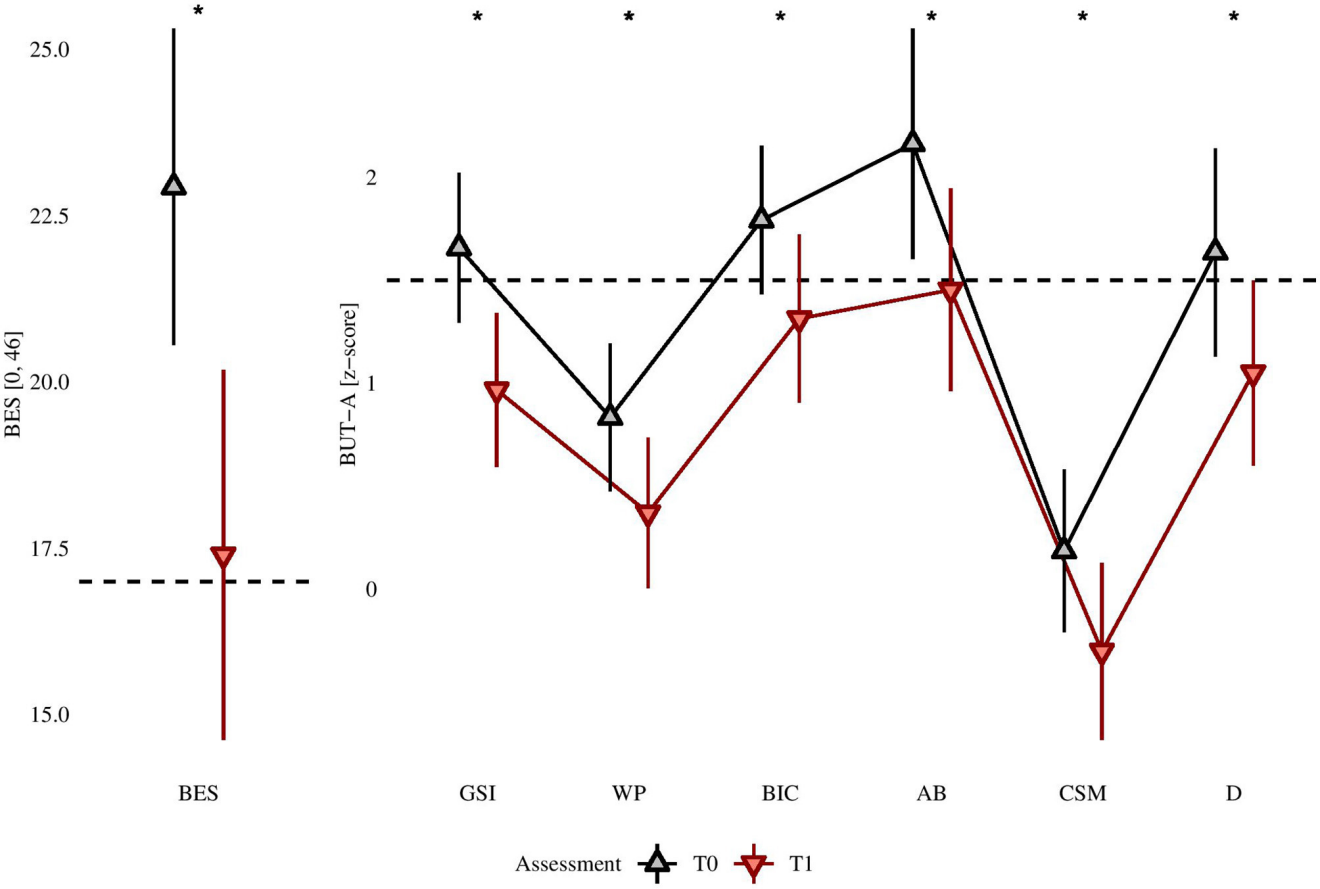
BES raw scores confronted between pre- and post-intervention assessment and BUT-A z- scores. ^*^Post treatment is lower than pre-treatment at statistically significant level, after Benjamini-Hochberg's correction, with *p* < 0.050.

**Figure 2:**
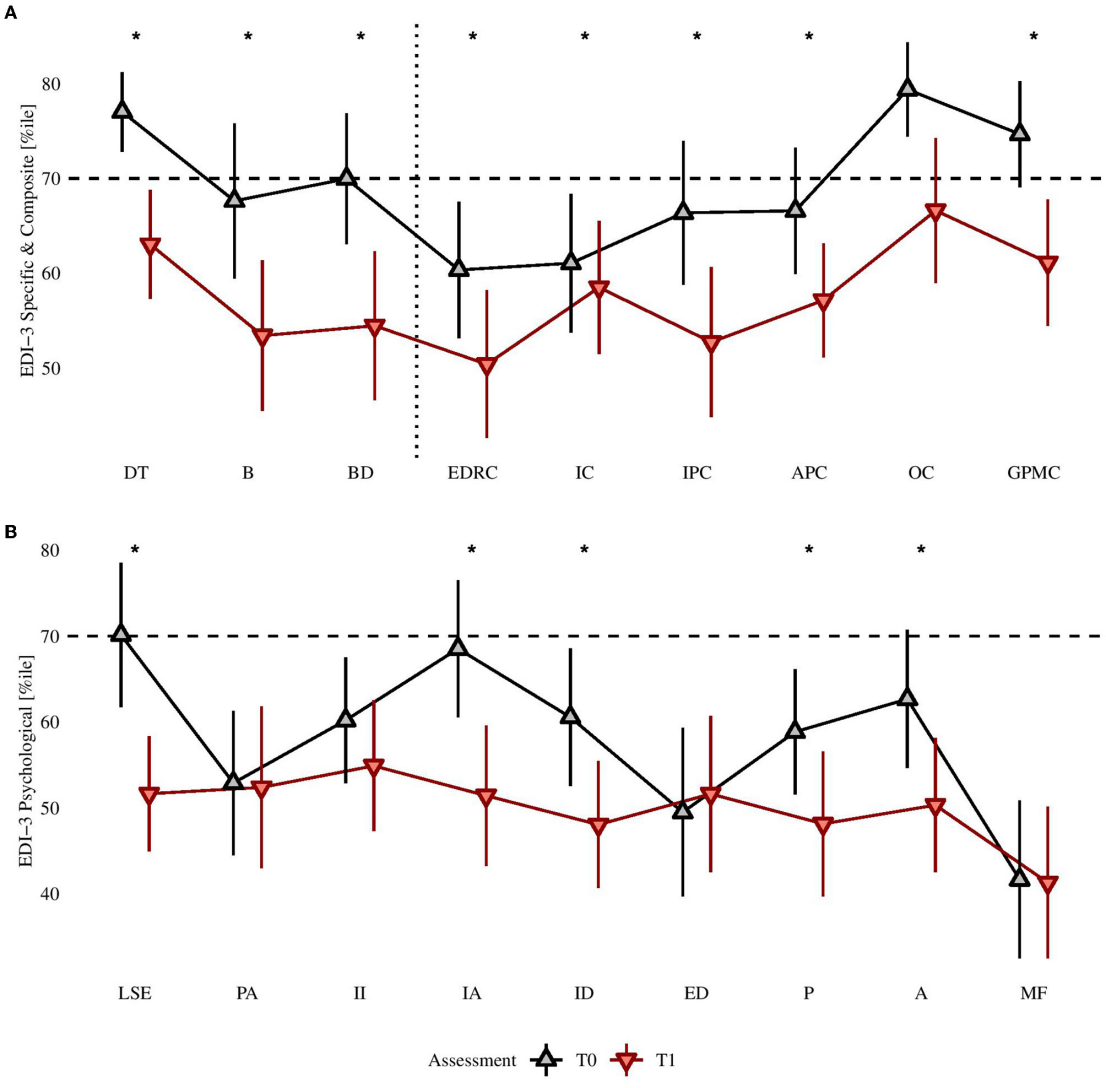
**(A)** EDI-3, Eating disorder specific and composite scores confronted pre- and post-treatment; **(B)** EDI-3 psychological scales confronted pre- and post-treatment. ^*^Post treatment is lower than pre-treatment at statistically significant level, after Benjamini-Hochberg's correction, with *p* < 0.050.

After treatment, all statistically significant differences were in the expected direction (i.e., showing a reduction in measures). The comparison of scores between pre-treatment and post-treatment assessments are shown in Table 3. After FDR correction for multiple comparisons, the 25 differences were still statistically significant, with: 11 medium-size effects (i.e., BES; EDI-3: B, BD, EDCR, IC, IPC, GPMC, LSE, IA; BUT-A: GSI; SCL-90-R: I-S) and 13 small-size effects (EDI-3: DT, APC, ID, P, A; BUT-A: WP, BIC, AB, CSM, D; SCL-90-R: TOT, DEP, ANX). The BMI reduction was instead of lower size (ΔBMI: 1.34 ± 2.166, 95% CI: [−2.08, 7.99]), although there was a reduction of the score in 71.1% of the sample. The frequency of BES scores above the cut-off decreased from the 82.2% to 53.3% (χ^2^(1) = +9.60, *p* = 0.002, without correction). The comparison of standardized scores with the available Italian normative samples are shown in Figures 1, 2. Considering EDI-3 scores, at pre- treatment the mean score was statistically significantly above a T-score of 70 (borderline level) in: B [*t*_(44)_ = +3.72, *p* < 0.001; *U* = 825.0, *p* < 0.001], BD [*t*_(44)_ = +1.64, *p* = 0.054; *U* = 691.0, *p* = 0.025], and EDRC [*t*_(44)_ = +3.30, *p* = 0.001; *U* = 794, *p* = 0.001]. At post-treatment, no scale was on average above the cut-off for borderline level symptoms. At pre-assessment, the A scale of BUT-A had mean scores above a z- score of 1.5 [*t*_(44)_ = +2.32, *p* = 0.013; *U* = 692.0, *p* = 0.025), while no scale was statistically significantly above this cut-off at the post assessment. In linear mixed-effects models, after FDR correction for multiple comparisons, the BMI reduction was statistically significant even after adjusting for obesity at pre-treatment [−0.95, *t*_(45)_ = −4.19, *p* < 0.001] and for other confounders.

**Table 3:** Pre-post (T0-T1) 12-weeks intervention comparison.

**Measure**			**T0**	**T1**	**Test**	**FDR-p**	**Effect size**
**Scale**			**Mean** **±SD**	**Mean** **±SD**	***t*_(44)_/U, *p***	***p***	**d [95% ci]**
BMI		[Kg/m^2^]	37.05 ± 7.181	35.71 ± 7.521	*t* = 4.1, *p* < 0.001	0.003**	0.180 [0.1, 0.3]^N^
BES		[0–46]	22.93 ± 8.142	17.40 ± 9.514	*t* = 4.5, *p* < 0.001	0.001**	0.620 [0.3, 0.9]^M^
EDI-3-S	DT	[%ile]	66.60 ± 22.815	57.16 ± 20.604	*t* = 3.2, *p* = 0.001	0.054	0.433 [0.2, 0.7]^S^
	B	[%ile]	79.40 ± 16.934	66.62 ± 26.171	*t* = 3.2, *p* = 0.001	0.049*	0.570 [0.2, 1.0]^M^
	BD	[%ile]	74.69 ± 19.178	61.13 ± 22.838	*t* = 3.8, *p* < 0.001	0.009**	0.640 [0.3, 1.0]^M^
EDI-3-C	EDRC	[%ile]	77.04 ± 14.338	63.04 ± 19.684	*t* = 5.3, *p* < 0.001	<0.001***	0.794 [0.5, 1.1]^M^
	IC	[%ile]	67.64 ± 27.973	53.42 ± 27.197	*t* = 3.7, *p* < 0.001	0.010*	0.515 [0.2, 0.8]^M^
	IPC	[%ile]	69.98 ± 23.580	54.47 ± 26.827	*t* = 4.0, *p* < 0.001	0.005**	0.612 [0.3, 0.9]^M^
	APC	[%ile]	60.36 ± 24.697	50.40 ± 26.649	*t* = 2.7, *p* = 0.005	0.204	0.387 [0.1, 0.7]^S^
	OC	[%ile]	61.07 ± 25.067	58.51 ± 24.098	*t* = 0.6, *p* = 0.265	1.000	0.104 [−0.2, 0.4]^N^
	GPMC	[%ile]	66.38 ± 25.885	52.73 ± 27.089	*t* = 3.4, *p* = 0.001	0.026*	0.515 [0.2, 0.8]^M^
EDI-3-P	LSE	[%ile]	70.13 ± 28.648	51.64 ± 22.890	*t* = 5.0, *p* < 0.001	<0.001***	0.703 [0.4, 1.0]^M^
	PA	[%ile]	52.89 ± 28.787	52.38 ± 32.234	*t* = 0.1, *p* = 0.451	1.000	0.017 [−0.3, 0.3]^N^
	II	[%ile]	60.20 ± 24.983	54.89 ± 26.053	*t* = 1.6, *p* = 0.056	1.000	0.208 [−0.1, 0.5]^S^
	IA	[%ile]	68.53 ± 27.304	51.42 ± 27.952	*t* = 3.5, *p* = 0.001	0.020*	0.619 [0.2, 1.0]^M^
	ID	[%ile]	60.56 ± 27.439	48.04 ± 25.234	*t* = 3.5, *p* = 0.001	0.024*	0.473 [0.2, 0.8]^S^
	ED	[%ile]	49.49 ± 33.549	51.62 ± 31.114	*t* = −0.54, *p* = 0.703	1.000	−0.066 [−0.3, 0.2]^N^
	P	[%ile]	58.84 ± 24.863	48.13 ± 28.855	*t* = 3.2, *p* = 0.001	0.047*	0.393 [0.1, 0.7]^S^
	A	[%ile]	62.67 ± 27.472	50.31 ± 26.761	*t* = 3.1, *p* = 0.002	0.067	0.456 [0.2, 0.8]^S^
	MF	[%ile]	41.64 ± 31.423	41.29 ± 30.237	*t* = 0.1, *p* = 0.462	1.000	0.012 [−0.2, 0.3]^N^
BUT-A	GSI	[0, 5]	2.24 ± 1.127	1.68 ± 1.088	*t* = 5.0, *p* < 0.001	<0.001***	0.507 [0.3, 0.7]^M^
	WP	[0, 5]	2.11 ± 1.148	1.68 ± 1.148	*t* = 2.9, *p* = 0.003	0.108	0.382 [0.1, 0.7]^S^
	BIC	[0, 5]	2.67 ± 1.071	2.24 ± 1.223	*t* = 3.6, *p* < 0.001	0.015*	0.364 [0.2, 0.6]^S^
	AB	[0, 5]	1.88 ± 1.320	1.42 ± 1.171	*t* = 3.8, *p* < 0.001	0.010**	0.364 [0.2, 0.6]^**S**^
	CSM	[0, 5]	1.16 ± 0.976	0.80 ± 0.961	*t* = 2.7, *p* = 0.005	0.188	0.367 [0.1, 0.7]^S^
	D	[0, 5]	1.53 ± 1.127	1.15 ± 1.061	*t* = 2.6, *p* = 0.006	0.234	0.347 [0.1, 0.6]^S^
BUT-B	PST	[0, 37]	14.99 ± 12.814	12.71 ± 13.329	*t* = 1.3, *p* = 0.109	1.000	0.174 [−0.1, 0.5]^N^
	PSDI	[0, 5]	1.97 ± 1.223	1.85 ± 1.464	*t* = 0.8, *p* = 0.219	1.000	0.093 [−0.1, 0.3]^N^
SCL-90-R	TOT	[0, 4]	1.11 ± 0.791	0.86 ± 0.758	*t* = 2.7, *p* = 0.005	0.180	0.330 [0.1, 0.6]^S^
	SOM	[0, 4]	1.15 ± 0.837	1.00 ± 0.836	*t* = 1.6, *p* = 0.055	1.000	0.190 [0.0, 0.4]^N^
	O-C	[0, 4]	1.19 ± 0.888	1.06 ± 0.941	*t* = 1.1, *p* = 0.129	1.000	0.142 [−0.1, 0.4]^N^
	I-S	[0, 4]	1.43 ± 0.986	0.86 ± 0.779	*U* = 728.0, *p* < 0.001	0.002**	0.626 [0.3, 0.9]^M^
	DEP	[0, 4]	1.13 ± 0.883	0.86 ± 0.793	*t* = 2.9, *p* = 0.003	0.121	0.310 [0.1, 0.5]^S^
	ANX	[0, 4]	1.19 ± 0.868	0.86 ± 0.748	*U* = 639.0, *p* = 0.004	0.137	0.410 [0.1, 0.7]^S^
	HOS	[0, 4]	0.70 ± 0.752	0.62 ± 0.758	*t* = 0.7, *p* = 0.234	1.000	0.104 [−0.2, 0.4]^N^
	PHOB	[0, 4]	0.51 ± 0.667	0.38 ± 0.564	*U* = 209.5, *p* = 0.105	1.000	0.201 [−0.1, 0.5]^S^
	PAR	[0, 4]	0.78 ± 0.719	0.75 ± 0.782	*t* = 0.4, *p* = 0.344	1.000	0.045 [−0.2, 0.3]^N^
	PSY	[0, 4]	0.52 ± 0.594	0.45 ± 0.636	*t* = 1.0, *p* = 0.156	1.000	0.110 [−0.1, 0.3]^N^
	SLEEP	[0, 4]	1.11 ± 1.002	1.23 ± 1.210	*t* = −0.7, *p* = 0.767	1.000	−0.110 [−0.4, 0.2]^N^

%ile, percentile; A, Ascetism (EDI-3); -A, BUT part A; AB, Avoidance Behaviors (BUT-A); ANX, Anxiety (SCL-90-R); APC, Affective Problems Composite (EDI-3); -B, BUT part B; B, Bulimia (EDI-3); BD, Body Dissatisfaction (EDI-3); BES, Binge Eating Scale; BIC, Body Image Concerns (BUT-A); BMI, body mass index (Kg/m^2^); BUT, Body Uneasiness Test; -C, Composite scores of EDI-3; ci, Confidence interval; CSM, Compulsive Self-Monitoring (BUT-A); d, Cohen's d; D, Depersonalization (BUT-A); DEP, Depression (SCL-90-R); DT, Drive for Thinness (EDI-3); ED, Emotional Dysregulation (EDI-3); EDI-3, Eating Disorder Inventory, version 3; EDRC, Eating Disorder Risk Composite (EDI-3); FDR, False Discovery Rate (Benjamini-Hochberg's correction); GPMC, Global Psychological Maladjustment (EDI-3); GSI, Global Severity Index (BUT-A); HOS, Hostility (SCL-90-R); IA, Interpersonal Alienation (EDI-3); IC, Ineffectiveness Composite (EDI-3); ID, Interoceptive Deficits (EDI-3); II, Interpersonal Insecurity (EDI-3); IPC, Interpersonal Problems Composite (EDI-3); I-S, Interpersonal Sensitivity (SCL-90-R); LSE, Low Self-Esteem (EDI-3); MF, Maturity Fears (EDI-3); O-C, Obsessive-Compulsive (SCL-90-R); OC, Overcontrol Composite (EDI-3); P, Perfectionism (EDI-3); -P, Psychological traits of EDI-3; PA, Personal Alienation (EDI-3); PAR, Paranoid Ideation (SCL-90-R); PHOB, Phobic Anxiety (SCL-90-R); PSDI, Positive Symptom Distress Index (BUT-B); PST, Positive Symptom Total (BUT-B); PSY, Psychoticism (SCL-90-R); -S, Specific scores of EDI-3; SCL-90-R, Symptom Checklist, 90-items, revised version; SD, Standard deviation; SLEEP, Sleep (SCL-90-R); SOM, Somatization (SCL-90-R); T0, First assessment (before intervention); T1, Second assessment (after 12-weeksintervention); TOT, Global Severity Index (SCL-90-R); WP, Weight Phobia (BUT-A). Statistically significant after correction, with p: <0.050 (*), <0.010 (**), <0.001 (***). Conventional effect size for d: |d| <0.2 (^N^: negligible), 0.2 ≤ |d| <0.5 (^S^: small), 0.5 ≤ |d| <0.8 (^M^: medium).

*Results of univariate analyses with control for independent multiple comparisons are also reported, together with an effect size estimation for paired samples*.

## Discussion

This study adds some evidence to support the validity of a psychoeducation treatment for BED, focusing on learning to manage interpersonal stress, increasing self-efficacy and self-esteem (14). Given the well-established importance of CBT in the treatment of BED, some preliminary remarks about the differences between our psychoeducational approach and that of CBT are necessary. Psychoeducation is a structured educational intervention that provides patients with knowledge about an illness and its treatment, integrating emotional and motivational aspects useful for coping with the illness and improving treatment adherence and effectiveness. Adapted CBT for eating disorders (CBT-E), on the other hand, is a psychotherapy that, while initially involving some psychoeducational interventions, is then aimed at activating processes of change in patients by confronting and questioning their ideas about weight, body image and nutrition and activating behaviors of better nutritional control. Another aspect that differentiates our GPT intervention is the length of treatment: group CBT-E typically lasts 20 sessions plus an initial diagnostic framing session (40), whereas GPT lasts only 12 sessions. Finally, CBT-E is an intervention managed by mental health professionals, whereas GPT is a multidisciplinary treatment including four interventions managed primarily by a dietitian in collaboration with mental health professionals. In summary, our multidisciplinary approach, which has similarities to treatments adopted by other research groups, has distinctive features compared to CBT-E.

In our study, the reduction found in the average BES score is in line with the results of a study that applied a similar therapeutic approach (15). Considering that the main scope of the intervention was to reduce the incidence and intensity of BED symptoms, achieving this with medium effect size, seems to support the effectiveness of the treatment.

Although small in magnitude, a significant reduction in mean BMI was also found after controlling for pre-treatment BMI. This can be considered an accessory result since weight loss was not a primary goal of the treatment. On the other hand, the literature emphasizes that even the most effective treatments for BED symptoms are unlikely to achieve significant results in weight reduction (41).

### ED Specific Measures

The GPT improved other specific dimensions of the eating disorder. Part A of the BUT, that measures specifically ED symptoms, showed a large reduction in the Global Severity Index (mean effect, *d* = +0.507 [+0.291, +0.723]), with several subscales (WP, BIC, AB, D, CSM) showing a smaller effect size. On the same level are the results from the analysis of the EDI-3 test: EDRC decreased on the post-treatment assessment, with a medium effect size. The same trend was found on the Bulimia and Body Dissatisfaction subscales, while the Thinness Drive showed a slightly smaller effect size. We argue that this effect can be seen as a consequence of learning to recognize the underlying mechanisms that trigger binges, lowering self-stigma and regaining a broader and more compassionate perspective on oneself (42). At least in part, these effects could be mediated by improvements in self-esteem levels (43), demonstrated by a medium-sized reduction in LSE score of the EDI-3. Part of the effect shown by this intervention might also be due to its ability to limit food related impulsivity, a recognized underlying mechanism if the disorder in people affected by BED, already reported by Schag et al. (44).

### Interpersonal Factors and General Psychopathology

One of the processes activated in the GPT was to stimulate the learning of assertive communication techniques and the reappraisal of negative emotions. The positive effect on these behaviors may have influenced the reduction achieved in the Interpersonal Problems Composite and in the Interpersonal Alienation of the EDI-3, as well as in the Interpersonal Sensitivity of the SCL-90-R. Finally, we can add a few thoughts on the effects of GPT on affective regulation. There is evidence in existing literature that BED population is more likely to suffer of psychiatric comorbidity, in particular anxiety and mood disorders (2, 45, 46). It has been argued that BED symptoms stem from maladaptive strategies of emotion regulation (22), which are also linked to the development and maintenance across psychopathology (47, 48). Therefore, a significant medium-sized effect in reducing scores of EDI-3 General Psychological Maladjustment Composite (GPMC) and a slightly smaller effect in Affective Problem Composite are promising results, considering that Emotion Regulation is one of the main topics included in this psychoeducation program. It should be noted that smaller sized reductions are registered also in TOT, ANX and DEP scores of SCL-90R, which could indicate efficacy in reducing general psychopathological burden in this sample.

### Study Limitations

In generalizing the results presented, it should be borne in mind that the study has a number of limitations. Firstly, the sample size is relatively small. For this reason, we have decided to discuss in detail only medium effects. A second limitation is the absence of a control group, an aspect that should be addressed in future research. Although the literature concerning BED has compared the efficacy of treatments with different theoretical and practical orientation (49), to our knowledge only the study by Wilfley et al. (8) and one by a research team of our institution directly compared a group treatment with psychoeducational approach with one of psychodynamic-interpersonal orientation (50). After that experience, we decided to develop the psychoeducational approach as a primary treatment for BED (14).

A third limitation is the absence of a follow-up evaluation. Given the small-to-medium size improvement achieved on the most significant measures detected, it would be important to understand whether these effects are maintained over time. In a disorder characterized by a chronic course, the indication should be to monitor the patients in order to verify their ability to follow what they have learnt, also to evaluate the possibility for a periodic repetition of the treatment.

## Conclusion

Our results suggest the potential of a GPT, organized by integrating psychoeducation and nutritional counseling (health education) in a group setting. The treatment proved to be efficacious and relatively cost-effective (if confronted with individual psychotherapy or longer group interventions) for BED, which is currently the most prevalent eating disorder in the general population and is linked to the development of high-burden health problems like type-2 diabetes, obesity and hypertension. It appears that GPT may be at least as acceptable to patients as the other treatments currently provided in this field.

We must acknowledge that this early evidence needs further, more in-depth research, which needs to include a control condition and a follow-up evaluation to increase the level of evidence supporting this kind of intervention. Future studies should include data demonstrating that the beneficial effects are maintained over time, leading to prolonged change and recovery for patients, and should be compared with treatments considered most effective in reducing the symptoms of BED.

## Data Availability Statement

The raw data supporting the conclusions of this article will be made available by the authors, without undue reservation.

## Ethics Statement

Ethical review and approval was not required for the study on human participants in accordance with the local legislation and institutional requirements. The patients/participants provided their written informed consent to participate in this study.

## Author Contributions

GF and MBE conducted the treatment and collected the data. SL and GF collected the data and wrote the draft of the paper. LS supervised the work of SL. MG did the analyses. MBA was the responsible, general supervisor of the project and of the final version of the paper. All authors contributed to the article and approved the submitted version.

## Conflict of Interest

The authors declare that the research was conducted in the absence of any commercial or financial relationships that could be construed as a potential conflict of interest.

## Publisher's Note

All claims expressed in this article are solely those of the authors and do not necessarily represent those of their affiliated organizations, or those of the publisher, the editors and the reviewers. Any product that may be evaluated in this article, or claim that may be made by its manufacturer, is not guaranteed or endorsed by the publisher.
